# Genotype Combinations and Genetic Risk Score Analyses of *MTHFR*, *MTRR*, and *MTR* Polymorphisms in Hypothyroidism Susceptibility: A Case–Control Study

**DOI:** 10.3390/cimb47100794

**Published:** 2025-09-25

**Authors:** Nilgun Tan Tabakoglu, Arzu Ay, Nevra Alkanli, Mehmet Celik

**Affiliations:** 1 Health Practice and Research Center, Faculty of Medicine Hospital, Trakya University, Edirne 22100, Turkey; 2 Department of Biophysics, Faculty of Medicine, Trakya University, Edirne 22100, Turkey; arzuay@trakya.edu.tr; 3 Department of Biophysics, Faculty of Medicine, Haliç University, Istanbul 34445, Turkey; nevraalkanli@halic.edu.tr; 4 Department of Internal Diseases (Endocrinology and Metabolism Diseases), Faculty of Medicine, Trakya University, Edirne 22100, Turkey; mehmetcelik@trakya.edu.tr

**Keywords:** hypothyroidism, folate metabolism, homocysteine, genotype combination analysis, genetic risk factors, genetic risk score

## Abstract

Hypothyroidism is a multifactorial endocrine disorder where genetic predisposition plays a significant role. The *MTHFR, MTRR*, and *MTR* genes influence thyroid hormone regulation via homocysteine remethylation and DNA methylation. This study examined associations between hypothyroidism and polymorphisms in *MTHFR (C677T*–rs1801133, *A1298C*–rs1801131), *MTRR (A66G*–rs1801394), and *MTR (A2756G*–rs1805087) genes. Eighty-six patients with hypothyroidism and 87 healthy controls were included. Genotyping was performed using PCR-RFLP. Post hoc analysis confirmed adequate statistical power (95% for *MTRR A66G*, 84.6% for *MTR A2756G*). The study adhered to STROBE guidelines. *MTHFR* polymorphisms showed no significant association when considered individually. However, the *MTRR A66G AA* genotype was significantly more frequent in patients and conferred a markedly increased disease risk (OR: 4.373; 95% CI: 2.174–8.797; *p* < 0.001), while the *MTR A2756G AG* genotype was also more prevalent among patients and associated with higher susceptibility (OR: 2.178; 95% CI: 1.156–4.104; *p* = 0.008). Genotype combination analysis revealed that *CT–AA* (OR = 6.898; 95% CI: 1.941–24.516; *p* = 0.001) and *AG–AA* (OR = 6.892; 95% CI: 1.494–31.797; *p* = 0.007) conferred high risk. Certain genotypes correlated with clinical features, including hypercholesterolemia, diabetes, and cardiovascular disease. *MTRR A66G and MTR A2756G* polymorphisms are associated with hypothyroidism and metabolic comorbidities, both individually and in genotype combinations. These findings underscore the value of multilocus genetic models for understanding thyroid disorders and support the potential role of genetic biomarkers in personalized risk assessment and early diagnosis. GRS analysis demonstrated that each additional risk allele increased hypothyroidism risk (OR = 1.58; 95% CI: 1.18–2.10; *p* = 0.0018), and the total score showed moderate predictive power (AUC = 0.665; *p* < 0.001).

## 1. Introduction

Hypothyroidism (HT) is one of the most common endocrine disorders characterized by slowed body metabolism [1,2]. HT often has an insidious onset and may present with mild symptoms such as fatigue, weight gain, intolerance to colds, and depression [3]. However, if left untreated, serious complications such as myxoedema and even death may develop [3]. HT itself is a symptom. It may occur due to autoimmune diseases, secondary to radiotherapy treatment to the neck region, due to thyroidectomy surgeries, or as a side effect of some drugs such as amiodarone and lithium [2]. Hashimoto’s thyroiditis, an autoimmune condition, is the leading cause of HT in regions with inadequate iodine intake [4]. In addition to all these reasons, the number of HTs caused by thyroid cancers is also relatively high. The report GLOBOCAN 2022 projects that 821,214 new cases of thyroid cancer and 47,507 thyroid cancer-related fatalities occurred globally in 2022; the average age-standardized incidence rate in women is considerably greater than in men (13.6 per 100,000 versus 4.6 per 100,000) [5].

Various risk factors such as hormone imbalances, family history of disease, smoking and alcohol use, obesity, nutritional deficiencies with folic acid deficiency, and genetic differences are effective in the emergence of thyroid diseases [6]. For these reasons, folic acid deficiency is important regarding genomic instability. This condition, caused by changes in DNA methylation and repair mechanisms, has been associated with low folic acid levels. Consequently, low folic acid levels can lead to various pathological conditions, such as thyroid disorders [7]. Additionally, in HT, increased glucose, triglyceride, and cholesterol levels have been observed due to disruptions in the metabolic pathways of these molecules, which depend on amino acids [8].

Methylenetetrahydrofolate reductase (MTHFR), methionine synthase reductase (MTRR), and methionine synthase (MTR) enzymes play an important role in regulating folic acid metabolism [9]. MTHFR enzyme is important in DNA methylation, which regulates gene expression and converts 5,10-methylenetetrahydrofolate to 5-methyltetrahydrofolate via folic acid [10]. The *MTHFR* gene, consisting of 11 exons, is localized on chromosome 1p36.3, and two common genetic variations have been identified in this gene [11]. The *C677T* polymorphism (rs1801133) is located in exon 4 of the *MTHFR* gene, with a genomic coordinate of chr1:11856378 (GRCh38). The A1298C polymorphism (rs1801131) is located in exon 7, with a genomic coordinate of chr1:11854476 (GRCh38) [12,13]. The *MTHFR C677T* variant is defined by a substitution at codon 222, where alanine is replaced with valine. The *MTHFR A1298C* gene variation is characterized by a glutamate/alanine amino acid change at position 429 [10]. As a result of genetic variations identified in the *MTHFR* gene, this transformation is prevented, and enzymatic activity decreases [14]. *MTRR* stimulates the remethylation of homocysteine to methionine, and this gene is localized on chromosome 5 (5p15.2-p15.3). The *MTRR A66G* gene variation occurring in this gene has been associated with decreased MTRR enzyme levels [15,16]. The A2756G genetic variant observed in the *MTR* gene is marked by a base transition from adenine to guanine [17]. Due to the *A2756G* variant of the *MTR* gene that encodes the MTR enzyme, increased serum homocysteine levels occur, and folic acid metabolism is altered. It has been reported that this imbalance in folic acid metabolism may lead to thyroid disorders such as HT [16]. Therefore, we aimed to determine the associations of specific genetic variants, including *MTHFR (C677T, A1298C)*, *MTRR A66G*, and *MTR A2756G*, with HT susceptibility. Thus, we aimed to identify genetic biomarkers that could be effective in the development of the disease, based on the relationships of these genetic variations in patients diagnosed with HT. Additionally, in our study, we evaluated the predictive ability of the genetic risk score (GRS), a measure of an individual’s genetic predisposition to a relevant phenotype. Derived from *MTHFR, MTRR,* and *MTR* gene polymorphisms to predict HT susceptibility and its association with clinical comorbidities in a sample of HT patients [18].

Based on the high rates of metabolic and cardiovascular comorbidities observed in HT, we hypothesised that patients with a high genetic burden would have a higher disease risk and show stronger associations with additional risk factors. Furthermore, we also evaluated the predictive power of GRS for HT without any clinical model.

## 2. Materials and Methods

### 2.1. Study Design and Participants

The study was conducted between November and December 2023. Patient recruitment and clinical data collection were carried out at Trakya University Faculty of Medicine Health Research and Application Center, while molecular analyses and genotyping procedures were performed at the Department of Biophysics, Faculty of Medicine, Trakya University. Our study obtained approval from the Trakya University Faculty of Medicine Non-Invasive Scientific Research Ethics Committee, with the ethics committee approval protocol code TÜTF-GOBAEK 2023/340. Signed informed consent forms were collected from the patient group diagnosed with HT and the healthy control group. This study was designed and reported in accordance with the STROBE (Strengthening the Reporting of Observational Studies in Epidemiology) guidelines (Supplementary File S5).

Our study was conducted with a total of 173 people, including 86 patients diagnosed with HT and 87 healthy controls. The patient group in our study consisted of individuals who had been diagnosed with Hashimoto’s thyroiditis for at least three years (*n* = 44) and were receiving continuous thyroid hormone replacement therapy due to the disease, as well as individuals who had undergone total thyroidectomy (*n* = 43), regardless of the surgical indication. Individuals diagnosed with malignancy or blood-clotting disorders were excluded from the study. All inclusion and exclusion criteria were applied prior to sample collection, and blood samples were obtained only from individuals who fully met the study criteria. The eligibility of participants and their medical histories were confirmed through hospital records and verified using the national electronic health record system (e-Nabız) [19].

Therefore, no participants were excluded after enrollment, and the total number of participants analyzed was 173. Patients diagnosed with Hashimoto’s thyroiditis, according to the 2012 American Thyroid Association (ATA) guidelines, were enrolled in the study [20]. To minimize bias, a single-blind design was applied. Genotyping was performed without knowledge of case or control status to reduce measurement bias. The control group consisted of healthy volunteers who had not been diagnosed with any thyroid disease, including HT or any malignancy. Routine blood samples containing ethylenediaminetetraacetic acid (EDTA) were used to determine the genotype distributions of *MTHFR (C677T, A1298C)*, *MTRR A66G*, *and MTR A2756G* gene variations. Genotyping quality control was ensured by assessing DNA concentration and purity with a Nanodrop spectrophotometer and agarose gel electrophoresis. Duplicate assays were performed for each sample, and concordance was confirmed. Known positive controls were included to validate accuracy.

### 2.2. DNA Isolation

Genomic DNA was extracted from peripheral blood samples obtained from individuals diagnosed with HT and healthy controls. A commercial DNA extraction kit facilitated the process. To assess the integrity and concentration of the isolated DNA, samples from both patient and control groups underwent evaluation via a Nanodrop spectrophotometer. Additionally, the DNA samples were visualized through electrophoresis on a 0.8% agarose gel.

**Figure 1:**
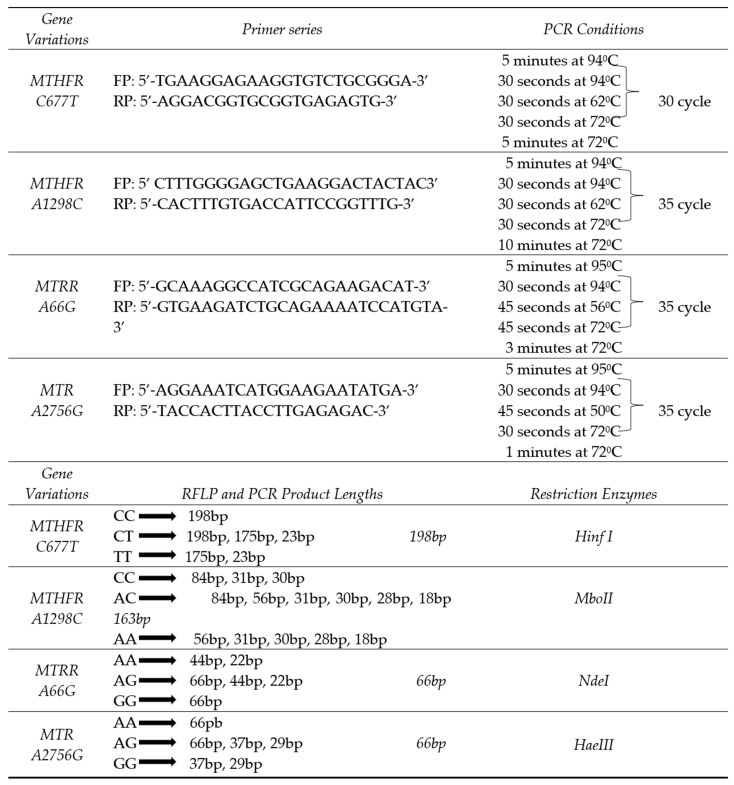
*MTHFR, MTRR, MTR* gene variations genotype distributions primer series, PCR conditions, PCR and RFLP components, product lengths, restriction enzymes; FP: Forward primer; RP: Reverse primer; PCR: Polymerase chain reaction.

The genotypic profiles of *MTHFR (C677T, A1298C), MTRR (A66G),* and *MTR (A2756G)* were assessed using polymerase chain reaction (PCR) and restriction fragment length polymorphism (RFLP) techniques. The amplified fragments generated through PCR and RFLP were visualized via electrophoresis using 2% and 3% agarose gels, respectively. Details, including genotype frequencies, primer sequences, fragment sizes, PCR parameters, and the specific restriction enzymes used, are summarized in Figure 1 (Figure S1–S4; Supplementary File S6).

### 2.3. Statistical Analysis

All statistical analyses were conducted using SPSS version 20.0 (IBM Corp., Armonk, NY, USA). The graphs and visualizations were created using Python 3.11. Pandas (2.2.2) and NumPy (1.26.4) were used for data processing; scikit-learn (1.5) for ROC curves and AUC calculations; statsmodels (0.14.2) and Matplotlib (3.8) for forest plots of logistic regression results; and Seaborn (0.13) for general visualization. The results are expressed as mean ± standard deviation or number (percentage), and a *p*-value < 0.05 was considered statistically significant. An independent samples t-test was used to compare the age of the hypothyroid patient and control groups. Logistic regression analysis was applied to evaluate categorical clinical parameters (hypertension, type 2 diabetes, dyslipidemia, cardiovascular conditions, alcohol use, and tobacco smoking), and odds ratios (ORs) with 95% confidence intervals were calculated.

To evaluate the independent association between each genetic polymorphism and HT, multivariate logistic regression analyses were conducted. Associations between polymorphisms and hypothyroidism were analyzed under a codominant model, with the homozygous wild-type genotype serving as the reference. Each genotype was included as a categorical predictor variable, with the homozygous wild-type genotype used as the reference category. ORs and 95% confidence intervals were calculated for each comparison. The models were adjusted for potential confounding variables, including diabetes mellitus, hypertension, hypercholesterolemia, cardiovascular disease, smoking, and alcohol consumption. Adjustment for these confounders was applied only in the analyses of genetic polymorphisms in relation to HT. A *p*-value of <0.05 was considered statistically significant.

Group-wise comparisons of genotype frequencies for the *MTHFR* (*C677T*, *A1298C*), *MTRR A66G*, and *MTR A2756G* polymorphisms were conducted using the Chi-square test. Also, genotype distributions were evaluated for conformity with the Hardy-Weinberg equilibrium (HWE) within the patient and control groups. Genotype–phenotype associations were assessed using Chi-square tests. Genotype combinations across *MTHFR*, *MTRR,* and *MTR* gene variants were analyzed, and logistic regression was used to estimate odds ratios for disease association. To control for multiple comparisons, *p*-values obtained from genotype combination analyses were adjusted using the Benjamini–Hochberg false discovery rate (FDR) correction.

The GRS was calculated by summing the number of risk alleles carried by each individual in the *MTHFR C677T (T allele)*, *MTHFR A1298C (C allele)*, *MTR A2756G (G allele)*, and *MTRR A66G (A allele)* polymorphisms, using the simple count method as described by Igo Jr et al. [21]. Each risk allele was assigned a point value, and the total GRS reflected the individual’s genetic predisposition. The distribution of GRS between hypothyroid patients and controls was assessed using the Mann–Whitney U test, as GRS values did not follow a normal distribution (Shapiro–Wilk test, *p* < 0.001). A binary logistic regression was then performed to assess the association between GRS (as a continuous variable) and HT status (case/control), with the significance threshold set at *p* < 0.05. The discriminative performance of the GRS was evaluated using ROC curve analysis (AUC with *p*-values). Additionally, GRS was categorized into low (0–2), medium (3–4), and high (5–6) risk groups based on quartile distributions. This stratification was guided by both the empirical distribution of GRS in the study population and its potential biological relevance. The association between GRS categories and HT was evaluated using contingency Tables and Fisher’s exact test. Odds ratios (OR) were calculated relative to the low GRS group (0–2).

An a priori sample size calculation based on Kvaratskhelia et al. (2020) indicated that at least 84 subjects per group (total *n* = 168) were required to achieve 80% power at α = 0.05. Our study included 173 participants (86 patients, 87 controls), thus meeting this requirement [22].

Post hoc power analyses were performed using G*Power version 3.1.9.4 to assess the adequacy of the sample size. Cohen’s w was calculated from chi-square statistics using the formula w = √(χ^2^/N), (N is the total sample size, χ^2^: Chi-square value) [23]. The *MTRR A66G* variant demonstrated a strong effect size (w = 0.325) with a statistical power of 95%, and *MTR A2756G* showed a moderate effect (w = 0.25) with 84.6% power. In contrast, *MTHFR C677T* (w = 0.052) and A1298C (w = 0.055) had petite effect sizes, resulting in insufficient statistical power (8.7% and 9.1%, respectively) to detect weak associations. Overall, post hoc analyses confirmed adequate power for the main findings. There was no missing data in the analyzed variables; all participants had complete genotype and clinical information.

HWE was assessed separately for four polymorphisms in the control group. Multiple comparison correction was applied using the Benjamini–Hochberg method for the significance level. This correction was applied only to HWE analyses, not to the genotype–HT association tests. Following the adjustment, the relationship between genotypes showing deviation from HWE and patient/healthy groups was assessed using the Pearson chi-square test. For the *MTRR A66G* polymorphism, which continued to show deviation from HWE, a sensitivity analysis was performed, excluding the most common heterozygous genotype (AG). The findings are interpreted within conventional significance thresholds but with consideration of the potential risk of Type I error.

## 3. Results

The distribution of patients with HT and the control group, categorized by GRS, along with their significance values, is shown in Table 1.

The risk of HT in individuals in the medium GRS group was approximately five times higher than in the low-risk group (OR: 5.00, 95% CI: 2.40–10.40, *p* < 0.0001). The high GRS group also carries a significantly increased risk (OR: 4.00, 95% CI: 1.39–11.49, *p* = 0.0138). These findings indicate a strong association between GRS and HT.

**Figure 2:**
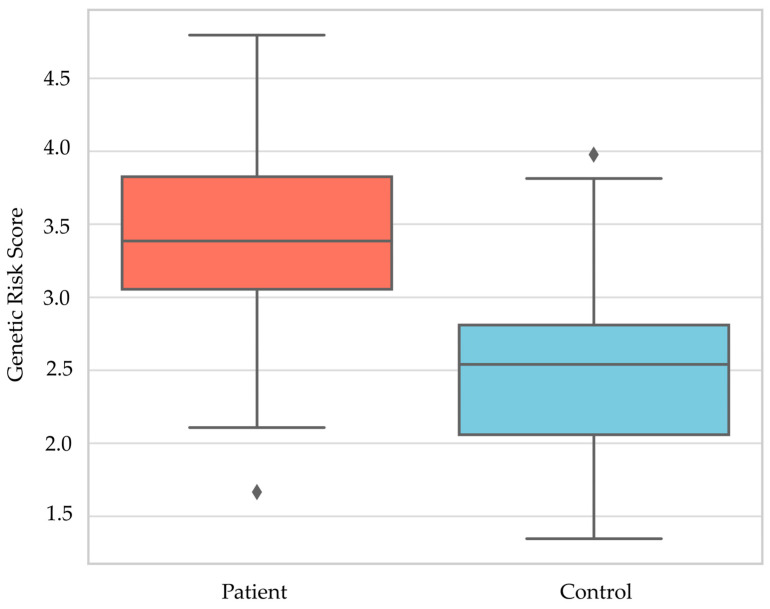
Genetic Risk Score Distribution; ♦, Outlier values.

The patient group exhibited a notably higher GRS compared to the control group (*p* = 0.0001, Mann–Whitney U test, Figure 2), reinforcing the hypothesis that HT is associated with an elevated genetic burden.

The outlier values shown in Figure 2 have not been excluded from the analysis; they are statistically expected extreme points.

**Figure 3:**
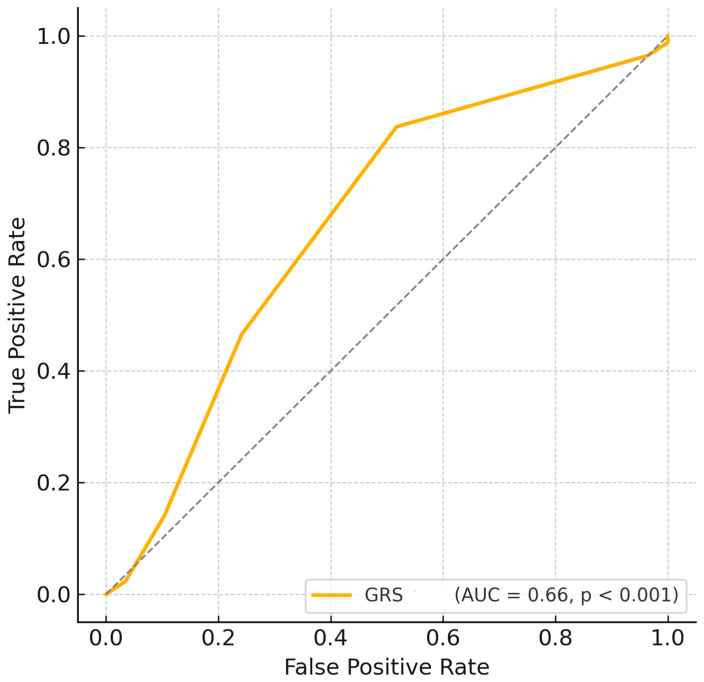
ROC curve based on Genetic Risk Score. GRS, Genetic risk score. -------, Random.

Logistic regression analysis further confirmed that GRS was independently associated with disease status (OR = 1.575, 95% CI: 1.184–2.095, *p* = 0.0018, Table 2), indicating that each additional risk allele increased the odds of having HT by approximately 57.5%. Furthermore, ROC curve analysis based solely on the GRS demonstrated a moderate predictive ability (AUC = 0.665, *p* < 0.001; Figure 3).

The evaluation of clinical and demographic variables (Table 3) demonstrated no statistically significant differences between individuals with HT and healthy controls regarding age and alcohol use (*p* > 0.05). However, the prevalence of conditions such as hypertension, type 2 diabetes, hypercholesterolemia, cardiovascular disorders, and tobacco use was notably higher among hypothyroid patients (all *p* < 0.05), suggesting a more substantial metabolic and cardiovascular comorbidity burden in individuals with HT.

Multivariate logistic regression analysis was performed to assess the association between each genetic polymorphism and HT, adjusting for potential confounders including diabetes, hypertension, cholesterol levels, heart disease, smoking, and alcohol use.

The *MTHFR A1298C* and *MTR A2756G* polymorphisms showed no significant association with HT after adjustment. However, a statistically significant association was observed for the *MTRR A66G* locus, where individuals with the AA genotype had a significantly higher risk of HT compared to the GG genotype (adjusted OR = 5.795; 95% CI: 1.708–19.665; *p* = 0.005; Table 4).

Regarding genotype distributions (Table 5), no significant differences were observed between groups for *MTHFR C677T* and *A1298C* polymorphisms (*p* > 0.05). Nevertheless, statistically significant correlations were observed for the *MTRR A66G* and *MTR A2756G* polymorphisms. The *AA* genotype of *MTRR A66G* was significantly more frequent in the hypothyroid group (OR: 4.373, 95% CI: 2.174–8.797, *p* < 0.001), while the *AG* genotype was more common in the control group (OR: 0.437, 95% CI: 0.237–0.806, *p* = 0.004). Similarly, for the *MTR A2756G* variant, the *AG* genotype was more prevalent in patients (OR: 2.178, 95% CI: 1.156–4.104, *p* = 0.008), whereas the *AA* genotype appeared protective, being significantly higher in controls (OR: 0.531, 95% CI: 0.290–0.974, *p* = 0.020).

The results imply that *MTRR A66G* and *MTR A2756G* variants could be involved in enhancing vulnerability to HT, while *MTHFR* variants alone may not contribute independently to disease risk.

**Figure 4:**
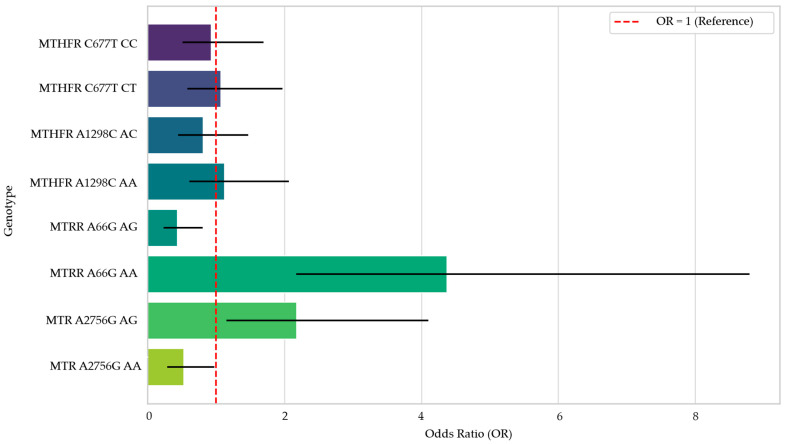
Forest Plot of Odds Ratios by Genotype.

The ratios with a 95% confidence interval for the evaluated genotypes are shown in Figure 4.

As shown in Table 6, HWE analysis revealed that the genotype distributions of *MTHFR C677T*, *MTHFR A1298C*, *MTRR A66G*, and *MTR A2756G* polymorphisms in the hypothyroid group conformed to HWE expectations (*p* > 0.05). However, significant deviations from HWE were observed in the healthy control group for *MTRR A66G* (*p* = 0.0038) and *MTR A2756G* (*p* = 0.0214).

In the control group, only the *MTRR A66G* genotype showed a significant HWE deviation after FDR correction (*p* = 0.0304). This variant was significantly associated with disease status (χ^2^ = 19.298, *p* = 0.0001). To test the robustness of this finding, a sensitivity analysis excluding the most common heterozygous genotype (*AG*) was conducted, which confirmed the association (χ^2^ = 10.991, *p* = 0.0009). Although *MTR A2756G* showed a nominal HWE deviation, it was not significant after correction (FDR, *p* = 0.0708) and was not included in sensitivity analyses.

Genotype distribution analyses according to clinical parameters (Table 7) revealed statistically significant associations for all examined gene variations (*MTHFR C677T, A1298C; MTRR A66G; MTR A2756G*) in the hypothyroid group (all *p* < 0.05). However, the Table presents only overall genotype counts across clinical subgroups, without detailing specific over- or under-representations within each comorbidity.

**Figure 5:**
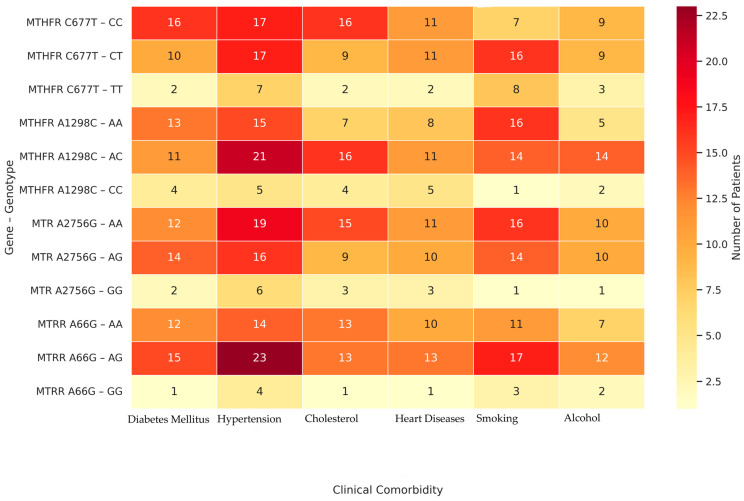
Heatmap of Clinical Comorbidities by Genotype;. The color scale represents the number of individuals carrying each genotype who presented with a given comorbidity. Darker red shades indicate higher patient counts, while lighter shades represent fewer individuals.

A detailed evaluation of Table 7 revealed genotype-specific patterns in the distribution of comorbidities among patients with hypothyroidism. Notably, the *AG* genotype of *MTRR A66G* was associated with the highest burden of comorbidities, including hypertension (*n* = 23), diabetes mellitus (*n* = 15), and cardiovascular disease (*n* = 13). Similarly, the AA genotype of *MTR A2756G* showed increased frequencies in patients with hypertension (*n* = 19) and hypercholesterolemia (*n* = 15), suggesting a possible role in vascular and lipid-related complications (Figure 5). In contrast, the TT genotype of *MTHFR C677T* and *CC* genotype of *MTHFR A1298C* were observed less frequently across all comorbidities, possibly indicating a protective effect.

Genotype combination analysis revealed several associations between gene variant combinations and HT susceptibility (Table 8). Initially, combinations such as *CC–AA* and *CT–AA* in the *MTHFR C677T/MTRR A66G* block, and *CC–AG and CT–AG* in the *MTHFR C677T/MTR A2756G* block showed statistically significant differences between patients and controls.

However, after applying the Benjamini–Hochberg FDR correction, only a subset of these associations remained statistically significant. Specifically, *CC–AA* and *CT–AA* retained significance, indicating a possible increased risk of HT (OR = 2.419 and OR = 6.898, respectively).

In contrast, *CC–AG* and *CT–AG (MTHFR C677T/MTR A2756G)* and *CC–GG (MTHFR C677T/MTRR A66G)*, while initially significant, did not remain significant after FDR adjustment, suggesting these findings should be interpreted with caution.

The *TT–AG* genotype combination was slightly more frequent in controls, although this difference did not reach statistical significance in either unadjusted or adjusted models.

Additional genotype combinations were also examined to assess multilocus effects on HT susceptibility. In the *MTHFR A1298C/MTR A2756G* combination, the *AC–AG* genotype combination remained significantly enriched in patients even after FDR correction, while the *AC–AA* and *AC–GG* combinations, though initially more common in controls, did not retain significance.

For *MTHFR A1298C/MTRR A66G*, the *AA–AA* and *AC–AA* genotype combinations showed robust associations with increased risk, both surviving FDR adjustment.

Likewise, in the *MTR A2756G/MTRR A66G* combination, *AA–AA, AG–AA*, and *AG–AG* remained significantly overrepresented in hypothyroid patients. However, *AA–GG,* despite being more frequent in controls, lost significance after correction.

These findings strengthen the evidence for the role of combined gene variant effects, particularly those involving *MTRR* and *MTR* loci, in modulating HT susceptibility.

## 4. Discussion

In this study, we observed significant associations between the *MTRR A66G* and *MTR A2756G* polymorphisms and an increased risk of HT. Although no significant differences were observed between patients and controls for the *MTHFR C677T* and *A1298C* polymorphisms, these findings should be interpreted with caution, as the small effect sizes suggest the study may have been underpowered to detect modest associations. In addition, our GRS analysis revealed that each additional risk allele significantly increased HT risk and that GRS alone demonstrated a moderate predictive ability for HT (AUC = 0.665, *p* < 0.001, Figure 3). This finding was consistent with the logistic regression results, and categorical analysis further demonstrated that the medium and high GRS groups were at a significantly increased risk (Table 1 and Table 2). Overall, our findings suggest that *MTR* and *MTRR* variants, together with the multilocus genetic burden measured by GRS, contribute to thyroid hormone regulation and HT risk.

The findings obtained in this study were generated using PCR-RFLP, a molecular analysis technique known for its high specificity and accuracy. The genotyping protocol applied enabled the precise identification of polymorphisms in the *MTHFR* (*C677T*, *A1298C*), *MTRR* (*A66G*), and *MTR* (*A2756G*) genes. The analysis was conducted using carefully designed primer sequences and appropriate restriction enzymes under optimized PCR cycling conditions, all of which are detailed comprehensively in Figure 1. This method provided a reliable foundation for genotyping, reinforcing the validity of the associations found between genetic variations and HT. Widely adopted in the literature, PCR-RFLP protocols are commonly used in analyzing genetic variations involved in folate metabolism, and the methodological framework employed in our study reflects this established analytical reliability [24].

Following genotyping, we observed statistically significant differences in the distribution of *MTRR A66G* and *MTR A2756G* polymorphisms between the hypothyroid patient group and healthy controls, as presented in Table 5. Specifically, the *AA* genotype of *MTRR A66G* was considerably more prevalent in patients (47.7%) compared to controls (17.2%), with an odds ratio of 4.373 (*p* < 0.001), indicating a strong association with disease susceptibility. This association remained significant even after adjustment for clinical covariates, as shown in Table 4 (adjusted OR = 5.795; 95% CI: 1.708–19.665; *p* = 0.005). In contrast, the *AG* genotype was more frequent in healthy individuals, suggesting a possible protective effect (OR, 0.437; *p* = 0.004). Similarly, the *AG* genotype of *MTR A2756G* was significantly enriched in the patient group (45.3%) relative to controls (27.6%) with an OR of 2.178 (*p* = 0.008), while the *AA* genotype was more dominant in controls (62.1%) and appeared to confer a lower risk (OR: 0.531, *p* = 0.020). These findings support the hypothesis that disruptions in genes involved in homocysteine remethylation and folate metabolism may play a role in thyroid hormone dysregulation. The literature has reported that the *MTRR A66G* polymorphism is associated with systemic diseases related to folate metabolism and certain autoimmune and metabolic disorders [25,26,27,28]. However, most of these studies do not directly address thyroid dysfunction; in contrast, our study indicates that this variation may also be associated with HT. In support of the broader relevance of these folate-pathway genes, a recent meta-analysis reported a significant association between the *MTR A2756G* polymorphism and male infertility. While *MTRR A66G* showed no effect in that context, its strong association with HT in our study suggests a gene- and tissue-specific pathogenic role within the same metabolic pathway [29].

The HWE assessment provided information on the distribution and genetic stability of the analyzed polymorphisms at the population level. In line with standard practice, HWE was evaluated based on observed and expected genotype distributions in the control group. In our study, the *MTHFR C677T* and *A1298C* polymorphisms were in HWE in both the patient and control groups (*p* > 0.05, Table 6), indicating no significant deviation from expected genotype frequencies and supporting the assumption of random mating for these loci. In contrast, *MTRR A66G* and *MTR A2756G* showed deviation in the control group (*p* = 0.0038 and *p* = 0.0214), but only *MTRR A66G* remained significant after FDR correction (FDR, *p* = 0.0304). To test whether this deviation affected the association, a sensitivity analysis was performed excluding the most common heterozygous genotype (*AG*) of *MTRR A66G*, and the association remained significant. This indicates that the HWE deviation did not significantly affect the observed association and that the deviation may stem from both population-specific factors and latent thyroid dysfunction in the control group.

Similar HWE deviations in control groups have been reported in studies involving metabolic or endocrine-related genes, highlighting the importance of cautious interpretation [30]. Despite these deviations, the overall consistency of HWE in the patient group strengthens the internal validity of our genotyping results and supports the observed associations between *MTRR A66G/MTR A2756G* polymorphisms and HT susceptibility. Importantly, all genotyping was repeated in duplicate, and concordant results were obtained, excluding laboratory error as a likely cause of these deviations.

In addition to assessing population-level genetic equilibrium, we examined whether the analyzed polymorphisms were associated with comorbid conditions frequently observed in hypothyroid patients. Recent large-scale studies have similarly reported that polygenic variants influence not only HT risk but also interact with metabolic and inflammatory parameters such as triglycerides, WBC count, and osteoporosis incidence [31].

Our analysis showed significant associations between certain genotypes and clinical features such as hypercholesterolemia, hypertension, diabetes, and cardiovascular disease (Table 7, *p* < 0.05). Figure 5 complements these findings by visualizing the distribution and comorbidity burden across genotype groups. Specifically, genotypes such as *MTRR A66G–AG* and *MTR A2756G–AA* are visually clustered with a higher frequency of multiple comorbidities, while *MTHFR C677T–TT* and *A1298C–CC* genotypes show minimal comorbidity presence, possibly indicating a protective profile. These associations may partly reflect the secondary effects of long-standing hypothyroidism, which alters lipid and glucose metabolism, vascular tone, and systemic inflammatory balance [8]. Moreover, the correlations observed for *MTRR A66G* and *MTR A2756G* with hypertension, smoking, and hypercholesterolemia may be amplified by the underlying hormonal milieu. Collectively, these findings suggest that certain genetic variants (particularly *MTRR A66G–AG* and *MTR A2756G–AA*) may shape a broader systemic risk profile in hypothyroid patients by modulating both thyroid function and metabolic–cardiovascular comorbidities. In other words, it demonstrates that genetic predisposition interacts with hormonal and metabolic systems and that genotype-based analyses are important for personalized treatment approaches. In line with our findings, a recent randomized controlled trial demonstrated that personalized supplementation with 5-methyltetrahydrofolate, pyridoxal-5′-phosphate, and methylcobalamin in individuals with *MTHFR, MTR,* and *MTRR* polymorphisms led to significant reductions in both homocysteine and LDL-C levels, particularly among homozygous minor allele carriers, thereby reinforcing the clinical relevance of these genotypes in cardiovascular risk management [32]. Thus, our results underscore the importance of gene-disease interplay in shaping metabolic risk profiles among individuals with thyroid disorders.

The molecular mechanisms underlying *MTHFR*-related metabolic disturbances should be considered to better contextualize these associations. The association between the *MTHFR C677T CC* genotype and clinical features such as hypercholesterolemia, diabetes, and cardiovascular disease—demonstrated in our data (Table 7)—can be biologically explained by impaired folate-dependent homocysteine metabolism [33,34]. Mutations in the *MTHFR* gene reduce the enzymatic efficiency of homocysteine remethylation, leading to elevated circulating homocysteine levels, known as hyperhomocysteinemia (HHcy), a well-established risk factor for vascular dysfunction [35]. Accumulated homocysteine disrupts endothelial function via multiple mechanisms, including enhanced reactive oxygen species (ROS) production, suppression of nitric oxide (NO)-mediated vasodilation, and the induction of pro-inflammatory cytokines such as IL-6 and TNF-α [34]. Additionally, Hcy impairs mitochondrial function by interfering with electron transport, reducing ATP synthesis, and promoting cellular damage through apoptosis and pyroptosis, as demonstrated in experimental models of myocardial ischemia–reperfusion injury [36]. These effects may be particularly detrimental in genetically predisposed individuals, exacerbating the metabolic and cardiovascular burden in the context of thyroid hormone deficiency. Accordingly, our findings align with known biochemical pathways and highlight the relevance of *MTHFR* polymorphisms in modulating systemic metabolic vulnerability in patients with HT. While this study identified statistically significant associations between *MTRR A66G* and *MTR A2756G* gene variants and HT, no significant differences were observed for the *MTHFR C677T* and *A1298C* polymorphisms. However, post hoc power analyses indicated that the non-significant results may have been due to the small observed effect sizes (w = 0.052 and w = 0.055, respectively), rather than the complete absence of an actual association. The statistical power for detecting these minor effects was found to be only 8.7% for *MTHFR C677T* and 9.1% for *A1298C,* well below the conventional threshold of 80%. Therefore, the nonsignificant outcomes should be interpreted with caution, as the sample size used in this study may not have been sufficient to capture subtle genetic influences of *MTHFR* polymorphisms on thyroid dysfunction. Future studies with larger sample sizes and meta-analytic approaches may help clarify the role of *MTHFR* variants in the pathophysiology of HT. While our study did not find statistically significant associations for *MTHFR* variants, this may reflect limited sample power rather than the absence of an actual effect. Supporting this possibility, a recent meta-analysis demonstrated a significant association between the *C677T* polymorphism and increased HT risk while suggesting a potential protective role for *A1298C* [37].

In light of these genotype–phenotype associations, we further examined how these clinical comorbidities were distributed between the patient and control groups.

As shown in Table 3, hypothyroid patients had markedly higher rates of hypertension (36.1% vs. 11.5%), diabetes (24.4% vs. 8.1%), hypercholesterolemia (27.9% vs. 3.4%), heart disease (24.4% vs. 3.4%), and smoking (25.6% vs. 10.3%) compared to controls, with all differences reaching statistical significance (*p* < 0.01). The calculated odds ratios revealed powerful associations for hypercholesterolemia (OR: 10.839) and heart disease (OR: 9.046), suggesting that HT may be associated with a greater burden of metabolic and cardiovascular comorbidities [38]. These findings are consistent with the known systemic impact of chronic thyroid hormone deficiency, which contributes to hypercholesterolemia, insulin resistance, and increased cardiovascular risk [39,40]. The high prevalence of smoking in the patient group may further exacerbate these metabolic disturbances, reinforcing the importance of evaluating lifestyle factors in hypothyroid individuals. While some differences, such as age and alcohol consumption, did not reach statistical significance, the overall trend supports a pattern of systemic metabolic vulnerability in the patient cohort.

Given the significantly higher prevalence of metabolic and cardiovascular comorbidities observed in hypothyroid patients (as demonstrated in Table 3), we further investigated whether combinations of genetic variants could better explain individual susceptibility to disease. Recent large-scale GWAS have identified over 130 genetic risk loci for HT, highlighting immune-related gene networks and providing a foundation for such investigations [41]. Therefore, genotype combination analysis was performed to explore potential gene–gene interactions among the studied polymorphisms, as previous studies have demonstrated that combinations of *MTHFR* gene variants, particularly *C677T* and *A1298C*, are significantly associated with comorbidities such as HT and congenital heart defects in genetically susceptible populations, including children with Down syndrome [42].

The results presented in Table 8 indicate that specific genotype combinations involving *MTHFR, MTR,* and *MTRR* gene variants are associated with HT; however, some associations lost their statistical significance after FDR correction. The *CT–AA (C677T/A66G)* and *AG–AA (A2756G/A66G)* genotype combinations were observed more frequently in the patient group and showed high odds ratios (OR = 6.898 and OR = 6.892); both remained statistically significant after adjustment. In contrast, combinations such as *CC–GG (C677T/A66G) AA–GG (A2756G/A66G)*, and *GG–AG (A2756G/A66G),* which initially appeared to have a protective effect (OR < 0.4), did not remain significant after FDR correction. Notably, the AA–AG (A2756G/A66G) genotype combination showed a protective effect (OR = 0.303) that remained statistically significant even after FDR correction.

These findings suggest that multilocus combinations may contribute to HT more substantially than individual polymorphisms alone, consistent with recent large-scale genomic studies demonstrating that aggregated genetic variants explain a significant proportion of variability in thyroid function and related disease risk [43]. This is biologically plausible, as these genes are functionally interconnected in the homocysteine–methionine cycle and collectively influence folate metabolism, DNA methylation, and redox homeostasis, as extensively reviewed in recent literature outlining the pleiotropic effects of *MTHFR* polymorphisms across diverse disease mechanisms [33]. The observed genotype combination patterns underscore the value of polygenic models in elucidating thyroid disease mechanisms and support the use of genotype combination-based genetic risk assessments in future research and potential precision medicine approaches.

This study has certain limitations due to the exploratory nature of the research and the limited sample size. Therefore, the results should be viewed as preliminary and considered as generating hypotheses rather than providing definitive evidence. First, although post hoc power analyses confirmed sufficient statistical power for detecting medium to strong associations in *MTRR* and *MTR* variants, the overall sample size remains relatively limited. It may have been insufficient to detect genetic effects, particularly in the *MTHFR* polymorphisms. Second, the cross-sectional nature of this case–control study limits our ability to establish causal relationships between the studied gene polymorphisms and HT and related metabolic comorbidities. Longitudinal studies would be required to clarify temporal associations and validate these findings over time. Third, the study was conducted in a single-center Turkish population, which may limit the generalizability of the findings to other ethnic or geographic groups. Fourth, gender and Body Mass Index data were not available for the participants, limiting our ability to adjust the genetic associations for these potential confounders. This should be addressed in future, larger-scale studies. Finally, although conventional univariate statistical tests were appropriately applied in this study, they may not fully account for the complex interplay between multiple genetic and environmental factors. Future studies may benefit from multivariate modeling techniques or integrative approaches such as polygenic risk scoring and machine learning to uncover deeper patterns and interactions.

Multivariate logistic regression models were applied and adjusted for potential confounders; however, the limited sample size reduces the robustness of these analyses. Therefore, these findings should be considered exploratory and warrant confirmation in larger, multicenter cohorts.

## 5. Conclusions

This study identifies *MTRR A66G* and *MTR A2756G* gene polymorphisms as significantly more prevalent in HT susceptibility in the Turkish population. While *MTHFR* gene variations (*C677T* and *A1298C*) did not individually correlate with HT risk, their involvement became more apparent in genotype combinations with *MTR* and *MTRR*. Specifically, genotype combinations such as *CT–AA* and *AG–AA* exhibited a strong association with disease occurrence, suggesting that multilocus interactions may more accurately reflect genetic predisposition than single polymorphisms alone. Furthermore, the genotype–phenotype correlations observed with metabolic comorbidities—particularly hypercholesterolemia, diabetes, and cardiovascular disease—highlight the systemic consequences of these genetic variations. These results underscore the complex interplay between folate metabolism, homocysteine pathways, and thyroid function. Ultimately, the study supports using genotype combination-based genetic profiling as a promising tool for early detection, personalized monitoring, and targeted prevention strategies in thyroid disorders. In addition, the GRS analysis showed that each additional risk allele was significantly associated with increased HT susceptibility, while the overall score demonstrated moderate predictive power. These results suggest that GRS could be a useful polygenic tool for estimating individual disease risk, particularly when used alongside genotype interaction models. The findings obtained are exploratory and should be validated through advanced analyses and multicenter studies using models that statistically control for confounding effects.

## Figures and Tables

**Table 1:** Patient and Control Distribution According to GRS Categories.

GRS Range	GRS Category	Patient Group	Control Group	Total	OR (Patient)	*p*-Value
0–2	Low	14	42	56	Reference	-
3–4	Medium	60	36	96	5.00	0.0001 *
5–6	High	12	9	21	4.00	0.0138 *

*, Fisher’s exact test; GRS, Genetic risk score.

**Table 2:** Logistic Regression Analysis of the Association Between Genetic Risk Score and Hypothyroidism Status.

Variable	OR: 95% Confidence Interval	*p*
Intercept	-	0.0028
Genetic Risk Score	1.575; 1.184–2.096	0.0018

**Table 3:** Comparison of Clinical and Demographic Parameters between Patients with Hypothyroidism and Healthy Control Groups.

Clinical and Demographic Parameters	Patient Group (*n* = 86)	Control Group (*n* = 87)	OR: 95% Confidence Interval	*p*
Age	54.779 ± 10.998	56.379 ± 11.221	1.600; 1.091–4.292	0.242 ^a^
Hypertension (+)	31 (36.1%)	10 (11.5%)	4.340; 1.965–9.585	<0.001 ^b^
Diabetes Mellitus (+)	21 (24.4%)	7 (8.1%)	3.692; 1.478–9.227	0.003 ^b^
Cholesterol (+)	24 (27.9%)	3 (3.4%)	10.839; 3.123–37.615	<0.001 ^b^
Heart Diseases (+)	21 (24.4%)	3 (3.4%)	9.046; 2.586–31.647	<0.001 ^b^
Alcohol (+)	13 (15.1%)	8 (9.2%)	1.759; 0.689–4.486	0.119 ^b^
Smoking (+)	22 (25.6%)	9 (10.3%)	2.979; 1.282–6.922	0.006 ^b^

^a^ Independent Samples Test. ^b^ Logistic Regression. (+): Available.

**Table 4:** Adjusted Odds Ratios (OR) for Genetic Polymorphisms in Relation to Hypothyroidism.

Polymorphism	Genotype (Reference)	OR: 95% Confidence Interval	*p*
MTHFR A1298C	AC vs. AA	0.689; 0.327–1.452	0.327
	CC vs. AA	1.056; 0.325–3.429	0.928
MTRR A66G	AA vs. GG	5.795; 1.708–19.665	0.005
	AG vs. GG	1.268; 0.406–3.960	0.683
MTR A2756G	AA vs. GG	1.189; 0.333–4.250	0.790

Adjusted for: Diabetes Mellitus, Hypertension, Hypercholesterolemia, Cardiovascular Disease, Smoking, and Alcohol consumption.

**Table 5:** Logistic Regression Analysis, Odds Ratio Values of *MTHFR, MTRR, MTR* Gene Variations, Genotype Distributions for Hypothyroidism Patients and Healthy Control Groups.

Genotype Distributions		Patient Group (*n* = 86)	Control Group (*n* = 87)	*p*
*MTHFR* *C677T*	CC	40 (46.5%)	42 (48.3%)	0.971 ^a^
	CT	34 (39.5%)	33 (37.9%)	
	TT	12 (14.0%)	12 (13.8%)	
Genotype distributions		Patient group (*n* = 86)	Control group (*n* = 87)	*p*
*MTHFR A1298C*	CC	11 (12.8%)	9 (10.3%)	0.761 ^a^
	AC	40 (46.5%)	45 (51.7%)	
	AA	35 (40.7%)	33 (38.0%)	
Genotype distributions		Patient group (*n* = 86)	Control group (*n* = 87)	*p*
*MTRR* *A66G*	GG	6 (7.0%)	15 (17.2%)	<0.001 ^a*^
	AG	39 (45.3%)	57 (65.6%)	
	AA	41 (47.7%)	15 (17.2%)	
Genotype distributions		Patient group (*n* = 86)	Control group (*n* = 87)	*p*
*MTR* *A2756G*	GG	7 (8.2%)	9 (10.3%)	0.052 ^a^
	AG	39 (45.3%)	24 (27.6%)	
	AA	40 (46.5%)	54 (62.1%)	
Genotype distributions		Patient group (*n* = 86) and Control group (*n* = 87)		*p*
*MTHFR* *C677T*	CC	OR: 0.932 (0.513–1.692)		0.408 ^b^
	CT	OR: 1.070 (0.580–1.973)		0.414 ^b^
	TT	Reference		-
Genotype distributions		Patient group (*n* = 86) and Control group (*n* = 87)		*p*
*MTHFR A1298C*	CC	Reference		-
	AC	OR: 0.812 (0.447–1.474)		0.247 ^b^
	AA	OR: 1.123 (0.610–2.068)		0.355 ^b^
Genotype distributions		Patient group (*n* = 86) and Control group (*n* = 87)		*p*
*MTRR* *A66G*	GG	Reference		-
	AG	OR: 0.437 (0.237–0.806)		0.004 ^b*^
	AA	OR: 4.373 (2.174–8.797)		<0.001 ^b*^
Genotype distributions		Patient group (*n* = 86) and Control group (*n* = 87)		*p*
*MTR* *A2756G*	GG	Reference		-
	AG	OR: 2.178 (1.156–4.104)		0.008 ^b*^
	AA	OR: 0.531 (0.290–0.974)		<0.020 ^b*^

^a^ Chi-square test; ^b^ Logistic regression; OR: Odds ratio; CC: Cytosine-Cytosine; CT: Cytosine-Thymine; TT: Thymine-Thymine; AC: Adenine-Cytosine; GG: Guanine-Guanine; AG: Adenine-Guanine; AA: Adenine-Adenine; *: Significance (*p* < 0.05).

**Table 6:** Genotype Distributions, Allele Frequencies, and Hardy–Weinberg Equilibrium Results for *MTHFR, MTRR,* and *MTR* Variants in Patients with Hypothyroidism and Controls.

Gene Variations	Patient Group (*n* = 86)			Control Group (*n* = 87)		
	Allele	Case	Frequency	Allele	Case	Frequency
*MTHFR* *C677T*	C	114	0.6628	C	117	0.6724
	T	58	0.3372	T	57	0.3276
	Total	172	1.0000	Total	174	1.0000
	Hardy–Weinberg Equilibrium Test: Pearson chi2 = 1.1482 Pr = 0.2839 ^a*^			Hardy–Weinberg Equilibrium Test: Pearson chi2 = 1.6810 Pr = 0.1948 ^a^*		
*MTHFR A1298C*	C	62	0.3605	C	63	0.3621
	A	110	0.6395	A	111	0.6379
	Total	172	1.0000	Total	174	1.0000
	Hardy–Weinberg Equilibrium Test: Pearson chi2 = 0.0067 Pr = 0.9348 ^a^			Hardy–Weinberg Equilibrium Test: Pearson chi2 = 1.2464 Pr = 0.2642 ^a^		
*MTRR* *A66G*	G	51	0.2965	G	87	0.5000
	A	121	0.3075	A	87	0.5000
	Total	172	1.0000	Total	174	1.0000
	Hardy–Weinberg Equilibrium Test: Pearson chi2 = 0.6512 Pr = 0.4197 ^a^			Hardy–Weinberg Equilibrium Test: Pearson chi2 = 8.3793 Pr = 0.0038 ^a^*		
*MTR* *A2756G*	G	53	0.3081	G	42	0.2414
	A	119	0.6919	A	132	0.7586
	Total	172	1.0000	Total	174	1.0000
	Hardy–Weinberg Equilibrium Test: Pearson chi2 = 0.3476 Pr = 0.5555 ^a^			Hardy–Weinberg Equilibrium Test: Pearson chi2 = 5.2972 Pr = 0.0214 ^a^*		

^a^ Hardy–Weinberg Equilibrium test; *: Significance (*p* < 0.05).

**Table 7:** *MTHFR, MTRR,* and *MTR* gene variations genotype distributions according to clinical parameters in patients with hypothyroidism.

*MTHFR* *C677T*	DM	HT	CHOL	HD	SM	ALC	Total	*p*
*CC*	12	10	14	11	5	5	57	<0.001 ^a^*
*CT*	7	16	9	8	12	6	58	
*TT*	2	5	1	2	5	2	17	
Total	21	31	24	21	22	13	132	
*MTHFR* *A1298C*	DM	HT	CHOL	HD	SM	ALC	Total	*p*
*CC*	4	4	4	5	1	1	19	0.004 ^a^*
*AC*	9	16	14	9	9	8	65	
*AA*	8	11	6	7	12	4	48	
Total	21	31	24	21	22	13	132	
*MTRR* *A66G*	DM	HT	CHOL	HD	SM	ALC	Total	*p*
*GG*	1	1	0	1	2	1	6	0.038 ^a^*
*AG*	10	17	13	10	8	7	65	
*AA*	10	13	11	10	12	5	61	
Total	21	31	24	21	22	13	132	
*MTR* *A2756G*	DM	HT	CHOL	HD	SM	ALC	Total	*p*
*GG*	2	5	3	2	1	0	13	0.010 ^a^*
*AG*	11	13	8	8	10	8	58	
*AA*	8	13	13	11	11	5	61	
Total	21	31	24	21	22	13	132	

DM: Diabetes Mellitus; HT: Hypertension; CHOL: Cholesterol; HD: Heart Diseases; SM: Smoking; ALC: Alcohol; CC: Cytosine-Cytosine; CT: Cytosine-Thymine; TT: Thymine-Thymine; AC: Adenine-Cytosine; GG: Guanine-Guanine; AG: Adenine-Guanine; AA: Adenine- Adenine; ^a^ Chi-Square; *: Significance (*p* < 0.05).

**Table 8:** Genotype Combinations Analysis, Frequencies, Odds Ratio values, and Adjusted *p*-Values (FDR) for *MTHFR*, *MTRR*, and *MTR* Gene Variations in Patients With Hypothyroidism and Controls.

Genotype Analysis C677T/A1298C	Patients (*n* = 86)	Frequency (%)	Controls (*n* = 87)	Frequency (%)	OR: (95%Cl), *p*
CC-CC	9	10.4	8	9.2	OR: 1.154 (0.423–3.146), *p* = 0.389
CC-AC	24	27.9	21	24.1	OR: 1.216 (0.616–2.403), *p* = 0.286
CC-AA	10	11.6	12	13.8	OR: 0.822 (0.335–2.018), *p* = 0.335
CT-AC	19	22.1	26	30.0	OR: 0.665 (0.335–1.321), *p* = 0.122
CT-AA	12	14.0	11	12.6	OR: 1.120 (0.465–2.697), *p* = 0.400
TT-AA	12	14.0	9	10.3	OR: 1.405 (0.560–3.530), *p* = 0.234
Genotype analysis C677T/A2756G	Patients (*n* = 86)	Frequency (%)	Controls (*n* = 87)	Frequency (%)	OR: (95%Cl), *p*
CC-AG	25	29.1	15	17.2	OR: 1.967 (0.952–4.063), *p* = 0.033 *
CC-AA	17	19.8	27	31.0	OR: 0.548 (0.272–1.101), *p* = 0.045 *
CT-GG	2	2.3	6	7.0	OR: 0.321 (0.063–1.639), *p* = 0.086
CT-AG	18	20.9	9	10.3	OR: 2.294 (0.967–5.441), *p* = 0.030 *
CT-AA	17	19.8	18	20.7	OR: 0.944 (0.450–1.984), *p* = 0.440
TT-GG	1	1.2	3	3.5	OR: 0.329 (0.034–3.231), *p* = 0.170
TT-AA	6	6.9	9	10.3	OR: 0.650 (0.221–1.912), *p* = 0.217
Genotype analysis C677T/A66G	Patients (*n* = 86)	Frequency (%)	Controls (*n* = 87)	Frequency (%)	OR: (95%Cl), *p*
CC-AA	24	27.9	12	13.8	OR: 2.419 (1.120–5.227), *p* = 0.012 ^†^*
CC-AG	18	20.9	18	20.6	OR: 1.015 (0.487–2.114), *p* = 0.484
CC-GG	4	4.6	12	13.8	OR: 0.305 (0.094–0.986), *p* = 0.024 ^†^*
CT-AA	17	19.8	3	3.5	OR: 6.898 (1.941–24.516), *p* = 0.001 *
CT-AG	19	22.1	30	34.5	OR: 0.539 (0.274–1.058), *p* = 0.036 *
TT-AG	3	3.5	9	10.3	OR: 0.313 (0.082–1.200), *p* = 0.045 *
TT-GG	1	1.2	3	3.5	OR: 0.329 (0.034–3.231), *p* = 0.170
Genotype analysis A1298C/A2756G	Patients (*n* = 86)	Frequency (%)	Controls (*n* = 87)	Frequency (%)	OR: (95%Cl), *p*
CC-AA	9	10.5	6	7.0	OR: 1.578 (0.536–4.642), *p* = 0.204
CC-AG	3	3.5	3	3.5	OR: 1.012 (0.199–5.159), *p* = 0.494
AC-AA	15	17.4	27	31.0	OR: 0.469 (0.229–0.963), *p* = 0.020 ^†^*
AC-AG	25	29.1	9	10.3	OR: 3.552 (1.545–8.164), *p* = 0.001 *
AC-GG	2	2.3	9	10.3	OR: 0.206 (0.043–0.985), *p* = 0.024 ^†^*
AA-AA	19	22.1	21	24.1	OR: 0.891 (0.439–1.808), *p* = 0.375
AA-AG	13	15.1	12	13.8	OR: 1.113 (0.477–2.599), *p* = 0.402
Genotype analysis A1298C/A66G	Patients (*n* = 86)	Frequency (%)	Controls (*n* = 87)	Frequency (%)	OR: (95%Cl), *p*
AA-AA	18	20.9	5	5.7	OR: 4.341 (1.532–12.302), *p* = 0.003 *
AA-AG	14	16.3	26	29.8	OR: 0.456 (0.219–0.950), *p* = 0.018 ^†^*
AA-GG	3	3.5	2	2.3	OR: 1.536 (0.250–9.430), *p* = 0.321
AC-AA	17	19.8	6	7.0	OR: 3.326 (1.243–8.902), *p* = 0.008 *
AC-AG	21	24.4	33	37.9	OR: 0.529 (0.274–1.018), *p* = 0.028 *
AC-GG	2	2.3	7	8.0	OR: 0.272 (0.055–1.349), *p* = 0.055
CC-AA	6	7.0	3	3.5	OR: 2.100 (0.508–8.682), *p* = 0.153
CC-AG	4	4.6	3	3.5	OR: 1.366 (0.296–6.292), *p* = 0.345
CC-GG	1	1.2	2	2.3	OR: 0.500 (0.044–5.618), *p* = 0.287
Genotype analysis A2756G/A66G	Patients (*n* = 86)	Frequency (%)	Controls (*n* = 87)	Frequency (%)	OR: (95%Cl), *p*
AA-AA	29	33.7	15	17.2	OR: 2.442 (1.196–4.985), *p* = 0.007 *
AA-AG	14	16.3	34	39.1	OR: 0.303 (0.148–0.620), *p* < 0.0001 *
AA-GG	1	1.2	9	10.3	OR: 0.102 (0.013–0.823), *p* = 0.016 ^†^*
AG-AA	12	14.0	2	2.3	OR: 6.892 (1.494–31.797), *p* = 0.007 *
AG-AG	24	27.9	9	10.3	OR: 3.355 (1.455–7.736), *p* = 0.002 *
AG-GG	2	2.3	6	7.0	OR: 0.321 (0.063–1.639), *p* = 0.086
GG-AG	4	4.6	12	13.8	OR: 0.305 (0.094–0.986), *p* = 0.024 ^†^*

OR: Odds ratio; CI: Confidence interval CC: Cytosine-Cytosine; CT: Cytosine-Thymine; TT: Thymine-Thymine; GG: Guanine-Guanine; AG: Adenine-Guanine; AA: Adenine-Adenine; AC: Adenine-Cytosine; *: Significance (*p* < 0.05); ^†^*: Indicates haplotypes that lost statistical significance after FDR correction, despite unadjusted *p* < 0.05.

## Data Availability

Our study data contain personal information of patients and, therefore, are not available for sharing due to the ‘Personal Data Protection Law’ and ethical reasons.

## Supplementary Materials

The following supporting information can be downloaded at: https://www.mdpi.com/article/10.3390/cimb47100794/s1.
