# Supplementary material for: Genotype Combinations and Genetic Risk Score Analyses of MTHFR, MTRR, and MTR Polymorphisms in Hypothyroidism Susceptibility: A Case–Control Study
Source: Curr Issues Mol Biol. 2025 Sep 25;47(10):794. doi: 10.3390/cimb47100794 (PMC12562523; doi:10.3390/cimb47100794)
Supplement: Supplementary file 1 [file cimb-47-00794-s001.zip › Supplementary File S5 STROBE Statement.pdf]

STROBE Statement—Checklist of items that should be included in reports of ***case-control studies***

	Item No	Recommendation
**Title and abstract**	1	(*a*) Indicate the study's design with a commonly used term in the title or the abstract: ***The study design is clearly indicated as a case-control study in both the title and the abstract. (Page 1)***
		(*b*) Provide in the abstract an informative and balanced summary of what was done and what was found: ***Yes. The abstract provides a concise and balanced summary of the study design, methods, key findings, and conclusions.***
**Introduction**		
Background/rationale	2	Explain the scientific background and rationale for the investigation being reported: ***Yes. The scientific background and rationale for the investigation are clearly explained in the Introduction section, with references to relevant literature. (Pages 2–3)***
Objectives	3	State specific objectives, including any prespecified hypotheses: ***Yes. The specific objectives and prespecified hypotheses are clearly stated at the end of the Introduction section. (Page 3)***
**Methods**		
Study design	4	Present key elements of study design early in the paper: ***Key elements of the study design (observational, single-center, case-control) are presented at the beginning of the Methods section. (Page 4)***
Setting	5	Describe the setting, locations, and relevant dates, including periods of recruitment, exposure, follow-up, and data collection: ***It setting, locations, and relevant dates, including the recruitment period (November–December 2023) and data collection sites, are clearly described in the Methods section. (Page 4)***
Participants	6	(*a*) Give the eligibility criteria, and the sources and methods of case ascertainment and control selection. Give the rationale for the choice of cases and controls: ***Eligibility criteria, sources, and methods of case and control selection are clearly described. The rationale for choosing Hashimoto's thyroiditis and total thyroidectomy patients as cases and healthy volunteers as controls is also provided. (Pages 4–5)***
		(*b*) For matched studies, give matching criteria and the number of controls per case: ***Not applicable. This is an unmatched case-control study. No matching criteria were used. (Page 4)***
Variables	7	Clearly define all outcomes, exposures, predictors, potential confounders, and effect modifiers. Give diagnostic criteria, if applicable: ***All outcomes (hypothyroidism), exposures (gene polymorphisms), and clinical predictors (e.g., hypertension, diabetes, smoking) are clearly defined. Diagnostic criteria for hypothyroidism include a confirmed diagnosis of Hashimoto's thyroiditis or history of total thyroidectomy. (Pages 4–6)***
Data sources/ measurement	8*	For each variable of interest, give sources of data and details of methods of assessment (measurement). Describe comparability of assessment methods if there is more than one group: ***Sources of data and assessment methods are clearly described for all variables. Genotyping was conducted using PCR-RFLP from blood samples. Clinical data were obtained from medical records. The same procedures were applied to both cases and controls, ensuring comparability. (Pages 5–6)***
Bias	9	Describe any efforts to address potential sources of bias: ***A single-blind design was used to minimize measurement bias; genotyping was performed without knowledge***

		of case or control status. (Page 5)
Study size	10	Explain how the study size was arrived at: ***No a priori sample size calculation was performed, but post hoc power analysis using Cohen's w indicated sufficient statistical power for key findings. (Page 6)***
Quantitative variables	11	Explain how quantitative variables were handled in the analyses. If applicable, describe which groupings were chosen and why: ***Quantitative variables such as age were expressed as mean ± standard deviation and compared using independent samples t-tests. No categorization or transformation was applied. (Page 6)***
Statistical methods	12	(*a*) Describe all statistical methods, including those used to control for confounding: ***Statistical methods included t-tests, chi-square tests, and logistic regression to control for potential confounders such as hypertension and diabetes. Analyses were conducted using SPSS version 20.0. (Page 6)***
		(*b*) Describe any methods used to examine subgroups and interactions: ***Subgroup analyses were conducted within the hypothyroid group to assess genotype–phenotype associations using Chi-square tests. Haplotype analyses were also performed to examine gene–gene interactions. (Pages 6–9)***
		(*c*) Explain how missing data were addressed: ***No missing data were reported; all analyses were conducted on complete datasets. (Pages 5–6)***
		(*d*) If applicable, explain how matching of cases and controls was addressed: ***This was an unmatched case-control study, and no matching criteria were used. (Page 4)***
		(*e*) Describe any sensitivity analyses: ***Yes***. ***Sensitivity analyses were performed and results are presented in the Results section (Page 5)***
**Results**		
Participants	13*	(a) Report numbers of individuals at each stage of study—eg numbers potentially eligible, examined for eligibility, confirmed eligible, included in the study, completing follow-up, and analysed: ***All inclusion and exclusion criteria were applied before sample collection. Only participants who met all eligibility conditions were enrolled. The final sample included 173 individuals (86 cases and 87 controls), and no participants were lost or excluded after enrollment. (Page 4)***
		(b) Give reasons for non-participation at each stage: ***All inclusion and exclusion criteria were applied prior to sample collection, and blood samples were obtained only from individuals who fully met the study criteria. The eligibility of participants and their medical histories were confirmed through hospital records and verified using the national electronic health record system (e-Nabız). Therefore, no participants were excluded after enrollment, and the total number of participants analyzed was 173. (Page 4)***
		(c) Consider use of a flow diagram: ***A flow diagram was not used, as all eligibility criteria were applied before sample collection, and no participants were excluded or lost during the study. (Page 4)***
Descriptive data	14*	(a) Give characteristics of study participants (eg demographic, clinical, social) and information on exposures and potential confounders: ***Demographic and clinical characteristics of participants, including age, comorbidities, and lifestyle factors, are reported in Table 2. Genotypic exposures and potential confounders are detailed in Table 3. (Pages 7–8)***
		(b) Indicate number of participants with missing data for each variable of interest: ***There were no missing data for any variables of interest; all participants had complete demographic, clinical, and genotypic information. (Page 6)***

Outcome data	15*	Report numbers in each exposure category, or summary measures of exposure: ***The numbers of participants in each genotypic exposure category (e.g., AA, AG, GG) for all studied polymorphisms are reported in Table 3, along with corresponding odds ratios and confidence intervals. (Page 8)***
Main results	16	(*a*) Give unadjusted estimates and, if applicable, confounder-adjusted estimates and their precision (eg, 95% confidence interval). Make clear which confounders were adjusted for and why they were included: ***Unadjusted odds ratios and 95% confidence intervals for each genotype were reported in Table 3. Logistic regression was applied to assess associations with clinical parameters, but genotypic estimates were not adjusted for confounders. (Page 8)***
		(*b*) Report category boundaries when continuous variables were categorized: ***No continuous variables were categorized; all continuous data (e.g., age) were analyzed as continuous variables using appropriate statistical methods. (Page 6–7)***
		(*c*) If relevant, consider translating estimates of relative risk into absolute risk for a meaningful time period: ***Not applicable. This was a case-control study without follow-up, and absolute risks over time could not be estimated. (Design limitation)***

Other analyses	17	Report other analyses done—eg, analyses of subgroups and interactions, and sensitivity analyses: ***Subgroup analyses were performed within the hypothyroid group to assess genotype–phenotype associations (Table 8). Genotype Combinations analyses were conducted to explore gene–gene interactions (Table 9). (Pages 8–9)***
**Discussion**		
Key results	18	Summarise key results with reference to study objectives: ***The Discussion section begins by summarizing the key findings in relation to the study objectives, emphasizing the association of MTRR A66G and MTR A2756G with hypothyroidism. (Page 9)***
Limitations	19	Discuss limitations of the study, taking into account sources of potential bias or imprecision. Discuss both direction and magnitude of any potential bias: ***The study limitations are clearly discussed, including small sample size, cross-sectional design, and limited generalizability. The potential for underpowered analyses for MTHFR polymorphisms is acknowledged, addressing both the direction and magnitude of potential bias. (Page 11)***
Interpretation	20	Give a cautious overall interpretation of results considering objectives, limitations, multiplicity of analyses, results from similar studies, and other relevant evidence: ***The results are interpreted cautiously in light of the study objectives, limitations, and supporting evidence from similar studies. The potential influence of multiple comparisons and sample size constraints is acknowledged. (Pages 10–11)***
Generalisability	21	Discuss the generalisability (external validity) of the study results: ***The generalisability of the findings is addressed by acknowledging that the study was conducted in a single-center Turkish population, which may limit external validity to other ethnic or geographic groups. (Page 11)***
**Other information**		
Funding	22	Give the source of funding and the role of the funders for the present study and, if applicable, for the original study on which the present article is based: ***The study was funded by the Trakya University Scientific Research Projects Coordination Unit (TUBAP) under Project No: 2023/190. The funders had no role in the design, conduct, analysis, or reporting of the study. (Page 12)***

*Give information separately for cases and controls.

Note: An Explanation and Elaboration article discusses each checklist item and gives methodological background and published examples of transparent reporting. The STROBE checklist is best used in conjunction with this article (freely available on the Web sites of PLoS Medicine at http://www.plosmedicine.org/, Annals of Internal Medicine at http://www.annals.org/, and Epidemiology at http://www.epidem.com/). Information on the STROBE Initiative is available at http://www.strobe-statement.org.
