# Supplementary material for: Genotype Combinations and Genetic Risk Score Analyses of MTHFR, MTRR, and MTR Polymorphisms in Hypothyroidism Susceptibility: A Case–Control Study
Source: Curr Issues Mol Biol. 2025 Sep 25;47(10):794. doi: 10.3390/cimb47100794 (PMC12562523; doi:10.3390/cimb47100794)
Supplement: Supplementary file 1 [file cimb-47-00794-s001.zip › Supplementary File S6 PCR and RFLP Product Lengths.pdf]

PCR and RFLP Product Lengths:

For MTHFR C677T gene variation: CC: 198bp; CT: 198bp, 175bp, 23bp; TT: 175bp, 23bp. 23bp is not observed. PCR product length: 198bp.
Restriction Enzyme: Hinfl
Cutting Zone Recognition Sequence: GANTC (N represents any nucleotide)
Explanation: In the MTHFR C677T polymorphism, the C (cytosine) base at position 677 of the gene is changed to a T (thymine) base. This C>T change creates a recognition site for the Hinfl restriction enzyme (Figure 1).

For MTHFR A1298C gene variation: CC: 84bp, 31bp, 30bp, 28bp, 18bp; AC: 84bp, 56bp, 31bp, 30bp, 28bp,18bp; AA: 56bp, 31bp, 30bp, 28bp, 18bp.
30bp, 28bp, 18bp are not observed. PCR product length: 163bp.
Restriction Enzyme: MboII
Cutting Zone Recognition Sequence: GAAGANNNNNNN/N/ (N represents any nucleotide)
Explanation: In the MTHFR A1298C polymorphism, the A (adenine) base at position 1298 of the gene is converted to the C (cytosine) base. This A>C change affects the recognition site of the MboII restriction enzyme. Generally, the A allele (wild-type) contains the MboII cutting site, while the C allele (mutant) eliminates this cutting site (Figure 2).
https://link.springer.com/article/10.1007/s40200-022-01061-9

For MTRR A66G gene variation: GG: 66bp; AG: 66bp, 44bp, 22bp; AA: 44bp, 22bp. 22bp is not observed. PCR product length: 66bp.
Restriction Enzyme: NdeI
Cutting Zone Recognition Sequence: CA↓TATG
Explanation: In the MTRR A66G polymorphism, the A (adenine) base at position 66 of the gene is converted to the G (guanine) base. This A>G change affects the cutting site of the NdeI restriction enzyme (Figure 3).

For MTR A2756G gene variation: GG: 37bp, 29bp; AG: 66bp, 37bp, 29bp; AA: 66bp. 29bp is not observed. PCR product length: 66bp.
Restriction Enzyme: HaeIII
Cutting Zone Recognition Sequence: GG↓CC
Explanation: In the MTR A2756G polymorphism, the A (adenine) base at position 2756 of the gene is converted to G (guanine). This A>G change affects the recognition site of the HaeIII restriction enzyme (Figure 4).
https://link.springer.com/article/10.1007/s11033-022-07597-6
https://www.amjmedsci.org/article/S0002-9629(15)30457-2/abstract

MTHFR, MTRR, MTR rs numbers and gene locations:

MTHFR C677T Variation
Gene Name: MTHFR (Methylenetetrahydrofolate reductase)
Function: The MTHFR enzyme plays a critical role in folate metabolism. It converts 5,10-methylenetetrahydrofolate to 5-methyltetrahydrofolate, which serves as a methyl donor in the remethylation of homocysteine to methionine.
Genomic Location: The MTHFR gene is located at location 1p36.3 on the short arm of human chromosome 1. The C677T polymorphism is found in exon 4 of the gene.
Rs Number: rs1801133
Explanation: This polymorphism is the replacement of the cytosine (C) base with the thymine (T) base at nucleotide 677 of the MTHFR gene. This change results in the conversion of an alanine (Ala) amino acid to a valine (Val) amino acid at codon 222 (Ala222Val). As a result, the thermolability of this enzyme increases and its activity decreases.

MTHFR A1298C Variation
Gene Name: MTHFR (Methylenetetrahydrofolate reductase)
Function: It is located on the same gene as MTHFR C677T and similarly plays a role in folate and homocysteine metabolism.
Genomic Location: The MTHFR gene is located on chromosome 1p36.3. The A1298C polymorphism has been identified in exon 7 of the gene.
Rs Number: rs1801131
Explanation: This polymorphism is the replacement of adenine (A) with cytosine (C) at nucleotide 1298 of the MTHFR gene. This change results in a glutamate (Glu) amino acid change to alanine (Ala) at codon 429 (Glu429Ala). The A1298C polymorphism can also cause a decrease in enzyme activity, but is generally less effective than the C677T polymorphism.

MTRR A66G Variation
Gene Name: MTRR (Methionine Synthase Reductase)
Function: The MTRR enzyme catalyzes a reductive methylation reaction that is necessary for the maintenance of the activity of the methionine synthase (MTR) enzyme. Methionine synthase is the main enzyme that converts homocysteine to methionine.
Genomic Location: The MTRR gene is located on the short arm of human chromosome 5, at location 5p15.2-15.3.
Rs Number: rs1801394
Explanation: This polymorphism is the replacement of adenine (A) base with guanine (G) base in nucleotide 66 of the MTRR gene. This change leads to the conversion of an isoleucine (Ile) amino acid to methionine (Met) amino acid in codon 22 (Ile22Met). It is thought that this polymorphism may reduce the activity of methionine synthase by affecting its affinity with cobalamin (vitamin B12), the cofactor of the MTR enzyme.

MTR A2756G Variation
Gene Name: MTR (Methionine Synthase)
Function: The MTR enzyme (also known as 5-methyltetrahydrofolate-homocysteine methyltransferase) is the main enzyme that catalyzes the conversion of homocysteine to

methionine. This is a folate and vitamin B12 dependent reaction. Methionine is required for protein synthesis and other important methylation reactions.
Genomic Location: The MTR gene is located on human chromosome 10, but specific location details are not as widely reported as for the MTHFR and MTRR genes. It is registered in the NCBI Gene database with ID number 4548.
Rs Number: rs1805087
Explanation: This polymorphism is the replacement of the adenine (A) base with the guanine (G) base at nucleotide 2756 of the MTR gene. This change leads to the conversion of the amino acid aspartic acid (Asp) to the amino acid glycine (Gly) at codon 919 (Asp919Gly).

NCBI Gene (National Center for Biotechnology Information Gene): Provides comprehensive data on detailed information on genes, genomic locations, functions, and associated polymorphisms. It is the primary source for Rs numbers and gene locations.
MTHFR: https://www.ncbi.nlm.nih.gov/gene/4524- National Center for Biotechnology Information (NCBI). *Gene: MTHFR methylenetetrahydrofolate reductase [Homo sapiens (human)]*; Available online: https://www.ncbi.nlm.nih.gov/gene/4524 (accessed on 16 June 2025).
MTRR: https://www.ncbi.nlm.nih.gov/gene/4552
MTR: https://www.ncbi.nlm.nih.gov/gene/4548

dbSNP (Database of Single Nucleotide Polymorphisms): It contains detailed information of each rs number, genomic location, and allelic changes.
rs1801133 (MTHFR C677T): https://www.ncbi.nlm.nih.gov/snp/rs1801133 - National Center for Biotechnology Information (NCBI). SNP: rs1801133 (MTHFR C677T). Available online: https://www.ncbi.nlm.nih.gov/snp/rs1801133 (accessed on 16 June 2025).
rs1801131 (MTHFR A1298C): https://www.ncbi.nlm.nih.gov/snp/rs1801131
rs1801394 (MTRR A66G): https://www.ncbi.nlm.nih.gov/snp/rs1801394
rs1805087 (MTR A2756G): https://www.ncbi.nlm.nih.gov/snp/rs1805087
