# Supplementary figures and images for: Genotype Combinations and Genetic Risk Score Analyses of MTHFR, MTRR, and MTR Polymorphisms in Hypothyroidism Susceptibility: A Case–Control Study
Source: Curr Issues Mol Biol. 2025 Sep 25;47(10):794. doi: 10.3390/cimb47100794 (PMC12562523; doi:10.3390/cimb47100794)

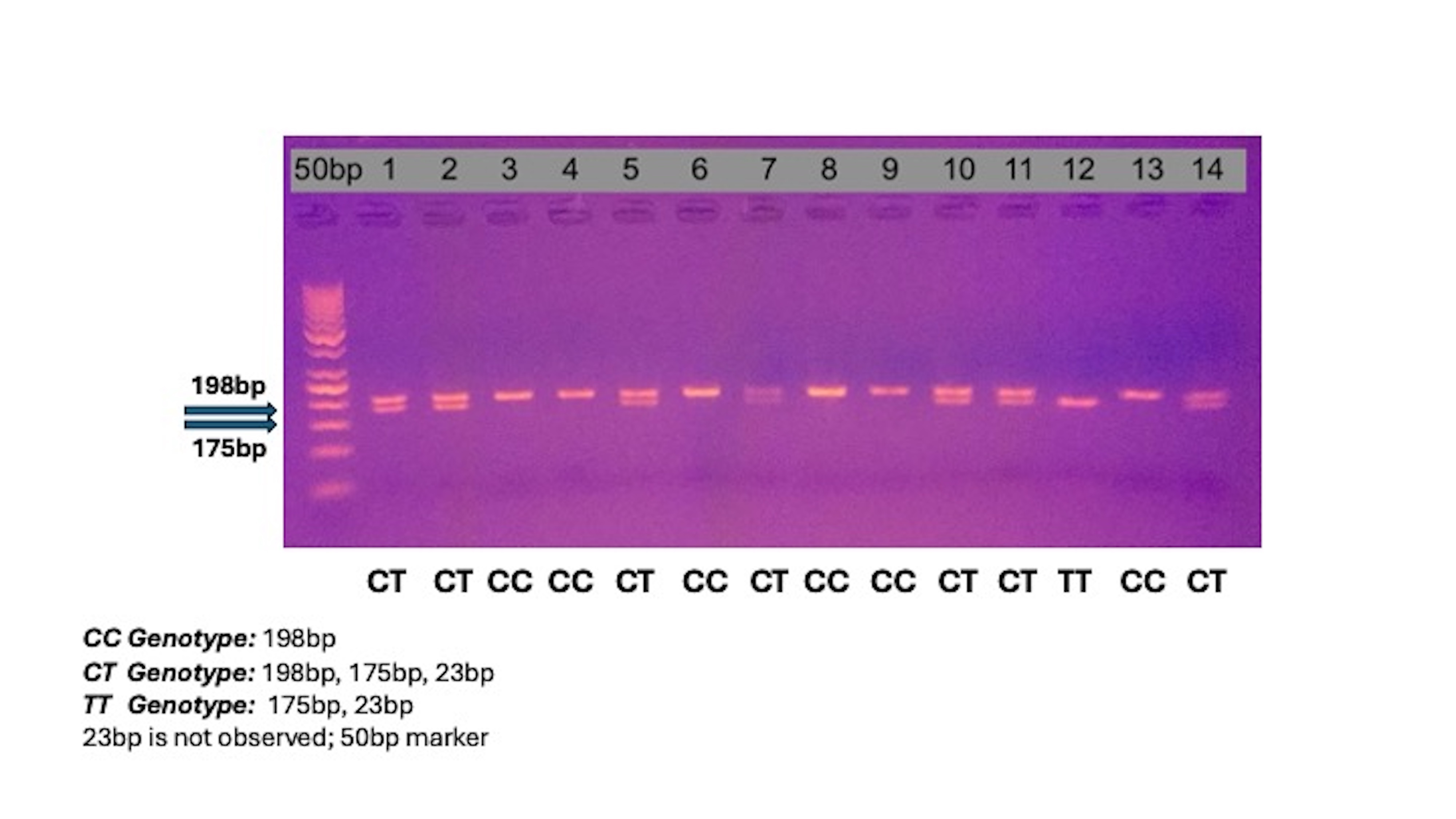

Supplement: Supplementary file 1 [file cimb-47-00794-s001.zip › Figure 1.jpg]

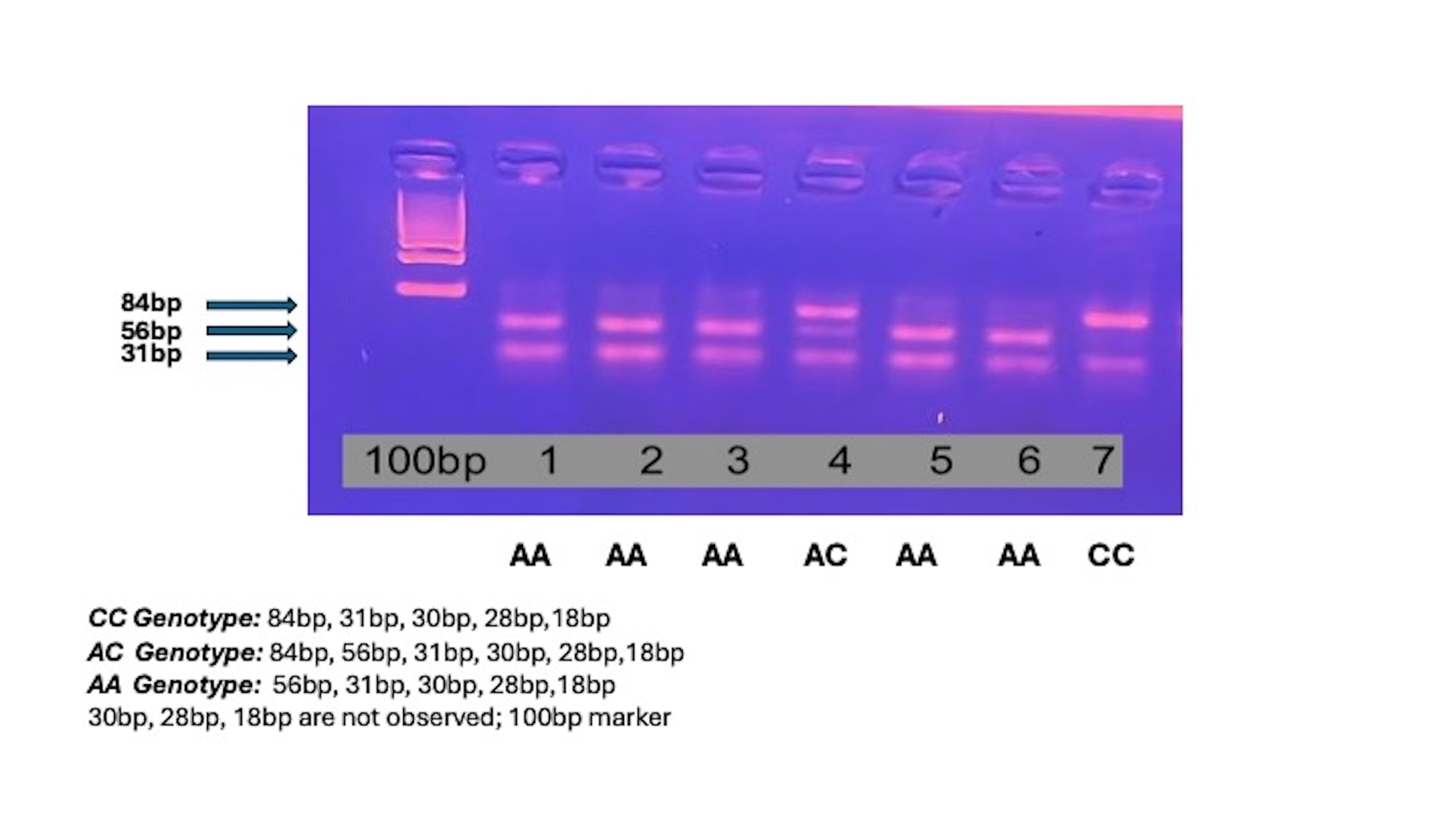

Supplement: Supplementary file 1 [file cimb-47-00794-s001.zip › Figure 2.jpg]

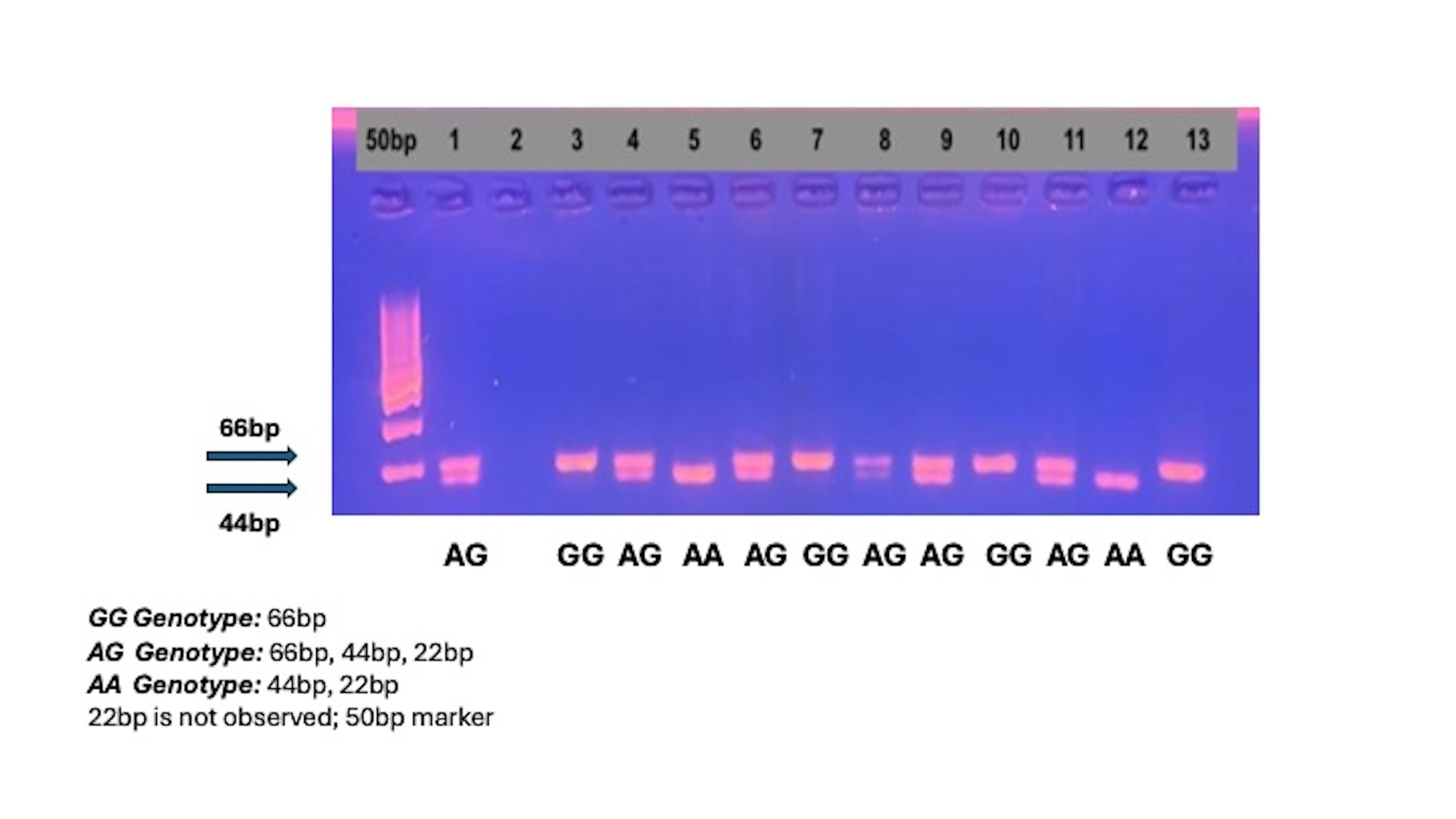

Supplement: Supplementary file 1 [file cimb-47-00794-s001.zip › Figure 3.jpg]

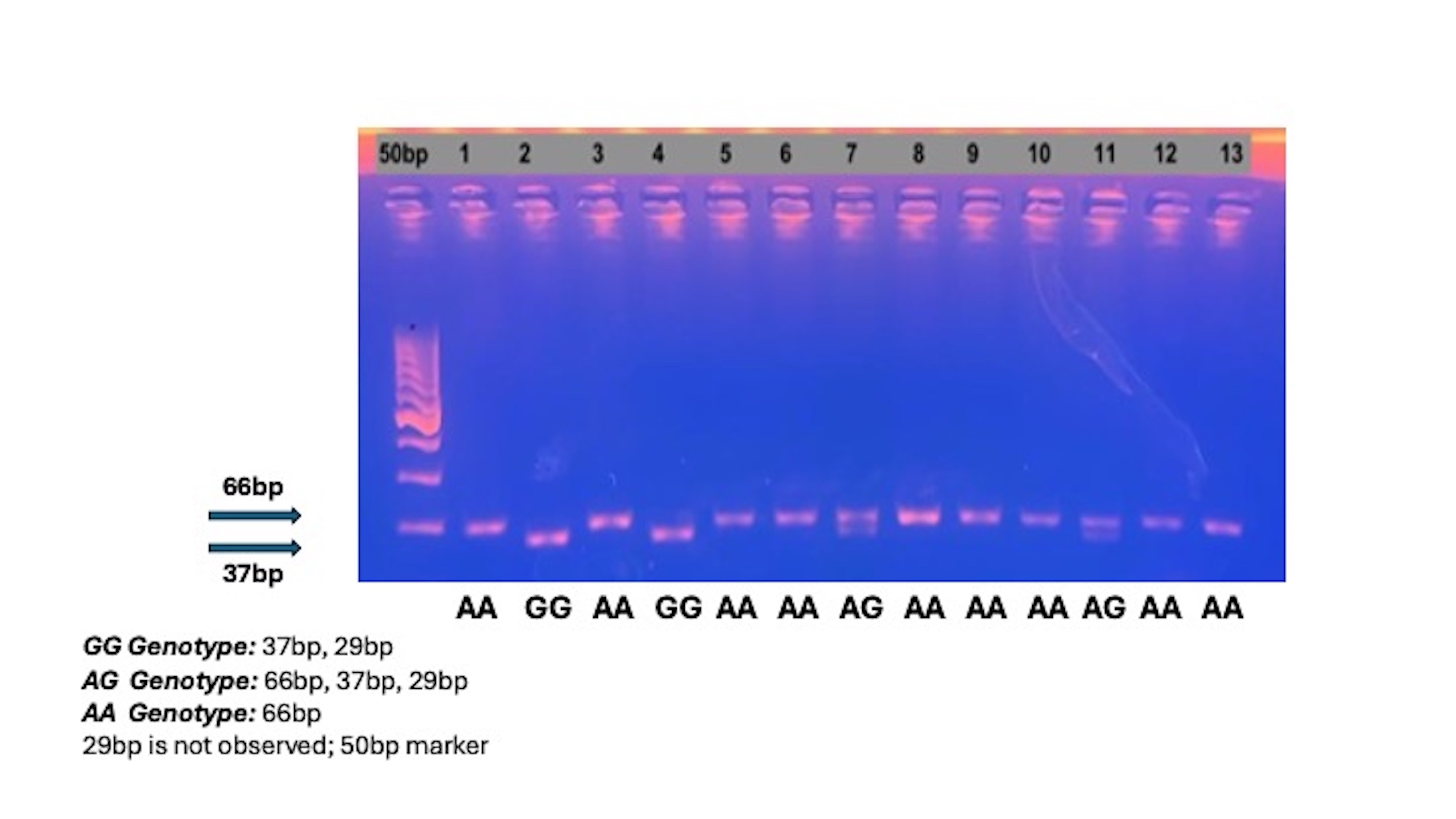

Supplement: Supplementary file 1 [file cimb-47-00794-s001.zip › Figure 4.jpg]
